# Development of a core outcome set for clinical trials in rosacea: study protocol for a systematic review of the literature and identification of a core outcome set using a Delphi survey

**DOI:** 10.1186/s13063-016-1554-3

**Published:** 2016-09-01

**Authors:** Sanjana Iyengar, Paula R. Williamson, Jochen Schmitt, Lena Johannsen, Ian A. Maher, Joseph F. Sobanko, Todd V. Cartee, Daniel Schlessinger, Emily Poon, Murad Alam

**Affiliations:** 1 Department of Dermatology, Feinberg School of Medicine, Northwestern University, 676 N. St. Clair St., Ste 1600, Chicago, IL 60611 USA; 2 Department of Biostatistics, University of Liverpool, Liverpool, UK; 3 Centre for Evidence-Based Healthcare, Medical Faculty Carl Gustav Carus, Technische Universität Dresden, Dresden, Germany; 4 Department of Dermatology, Saint Louis University School of Medicine, St. Louis, MO USA; 5 Department of Dermatology, University of Pennsylvania, Philadelphia, PA USA; 6 Division of Dermatologic Surgery, University of Pennsylvania, Philadelphia, PA USA; 7 Department of Dermatology, Penn State Hershey Dermatology, Hershey, PA USA; 8 Department of Otolaryngology, Feinberg School of Medicine, Northwestern University, Chicago, IL USA; 9 Department of Surgery, Feinberg School of Medicine, Northwestern University, Chicago, IL USA

**Keywords:** Core outcome set, Delphi, Consensus, Stakeholders, Rosacea, Systematic review

## Abstract

**Background:**

Rosacea is a chronic inflammatory disorder affecting millions of individuals worldwide. Diagnosis is based on signs and symptoms with management and treatment aimed to suppress inflammatory lesions, erythema, and telangiectasia. While many clinical trials of rosacea exist, the lack of consensus in outcome reporting across all trials poses a concern. Proper evaluation and comparison of treatment modalities is challenging. In order to address the inconsistencies present, this project aims to determine a core set of outcomes which should be evaluated in all clinical trials of rosacea.

**Methods/design:**

This project will utilize a methodology similar to previous core outcome set research. A long list of outcomes will be extracted over four phases: (1) systematic literature review, (2) patient interviews, (3) other published sources, and (4) stakeholder involvement. Potential outcomes will be examined by the Steering Committee to provide further insight. The Delphi process will then be performed to prioritize and condense the list of outcomes generated. Two homogenous groups of physicians and patients will participate in two consecutive rounds of Delphi surveys. A consensus meeting, composed of physicians, patients, and stakeholders, will be conducted after the Delphi exercise to further select outcomes, taking into account participant scores. By the end of the meeting, members will vote and decide on a final recommended set of core outcomes. For the duration of the study, we will be in collaboration with both the Core Outcome Measures in Effectiveness Trials (COMET) and Cochrane Skin Group - Core Outcome Set Initiative (CSG-COUSIN).

**Discussion:**

This study aims to develop a core outcome set to guide assessment in clinical trials of rosacea. The end-goal is to improve the reliability and consistency of outcome reporting, thereby allowing sufficient evaluation of treatment effectiveness and patient satisfaction.

**Electronic supplementary material:**

The online version of this article (doi:10.1186/s13063-016-1554-3) contains supplementary material, which is available to authorized users.

## Background

Rosacea is a chronic dermatologic disorder with a prevalence of about 2 %, affecting 16 million Americans [1, 2]. Fair-skinned individuals of Celtic and European heritage are most commonly affected, although all skin types may develop the condition [1, 3]. The etiology of rosacea is unknown and hence, diagnosis is often based on signs and symptoms [1]. Primary features of rosacea include inflammatory papules/pustules, erythema, and telangiectasia [4]. Secondary features consist of burning, plaques, and edema. Disease symptoms and lesions affecting the face and other visible areas may have a negative impact on health-related quality of life. Furthermore, patients with rosacea are at an increased risk for significant comorbidities such as autoimmune diseases, inflammatory bowel disease, and possibly cardiovascular disease [5–8].

In 2002, the National Rosacea Society Expert Committee classified rosacea into four main subtypes: erythematotelangiectatic, papulopustular, phymatous, and ocular [4, 9]. The erythematotelangiectatic subtype is the most common, with the phymatous subtype being rare. A variety of treatments, including topical, systemic, and laser therapies, have been utilized thus far. Topical treatments approved by the US Food and Drug Administration (FDA) include sodium sulfacetamide, ivermectin, azelaic acid, and metronidazole [1]. Subantimicrobial-dose doxycycline is the only FDA-approved systemic therapy for rosacea [1], but tetracycline and isotretinoin are often used as well [3]. Mirvaso (brimonidine tartrate) was approved in 2014 by the European Medicines Agency (EMA) to treat facial erythema caused by rosacea [10]. Laser therapy is recommended for the treatment of telangiectasia and rhinophyma.

Several trials are underway to evaluate new treatment modalities for rosacea. According to ClinicalTrials.gov, more than 100 trials relevant to rosacea are either in progress, actively recruiting, or complete [1, 11]. The 2015 Cochrane Review by van Zuuren assessed the efficacy and safety of interventions for rosacea with impact on quality of life [12]. Only 11 out of the 106 trials included in the review assessed the effect of rosacea treatment on quality of life, which made recommendations nearly impossible. Heterogeneity in physician-assessed secondary review outcomes, such as physician’s global evaluation, lesion counts, and duration of remission were pervasive in the trials. This inconsistency in outcomes measured across trials poses a concern when evaluating the effects of different interventions.

There are few validated methods of assessing improvement in rosacea, possibly due to the multiplicity of outcomes collected. Selective outcome reporting bias, defined as results-based selection of outcomes for publication, is a problem in many clinical trials and affects the conclusions of a significant proportion of systematic reviews [13].

Specific organizations have been formed to address outcome reporting bias. The Core Outcome Measures in Effectiveness Trials Initiative (COMET) brings together researchers interested in developing a standardized set of core outcomes in various health-related fields [14]. A core outcome set (COS) is defined as an agreed minimum set of outcomes that is recommended to be measured and reported in all clinical trials of a given condition or disease. The implementation of a COS may reduce the risk of selective outcome reporting and allow for more important outcomes to be assessed.

Another group, the Cochrane Skin Group - Core Outcome Set Initiative (CSG-COUSIN), was created specifically to address COSs in dermatology by examining outcome measures in current research [15]. CSG-COUSIN builds on the experiences of the Harmonizing Outcome Measures for Eczema (HOME) initiative, which also developed a roadmap to guide the process of COS development and implementation [16–21]. Similar to other initiatives, the CSG-COUSIN group hopes to develop standardized, evidence-based core outcome sets which can be utilized in all clinical trials.

While COS are under development for several dermatologic conditions, work has yet to be done to identify core outcomes specific for rosacea. In order to minimize duplication, this study has been registered with the COMET and CSG-COUSIN organizations so researchers are aware of our ongoing efforts and may participate if interested.

### Objective

The aim of this study is to develop an international COS that is relevant to clinical trials of rosacea based on the systematic synthesis of peer-reviewed research evidence; preferences from relevant stakeholder groups, including patients, clinicians, regulators; and structured consensus processes involving all relevant perspectives [21]. Objectives include first determining *what* should be measured and second, *how* it should be measured in rosacea clinical trials. The selected outcomes that may be recommended for inclusion in all rosacea research trials will be a minimum set that will not preclude inclusion of other outcomes in specific trials, per the preference of individual investigators..

## Methods/design

**Fig. 1:**
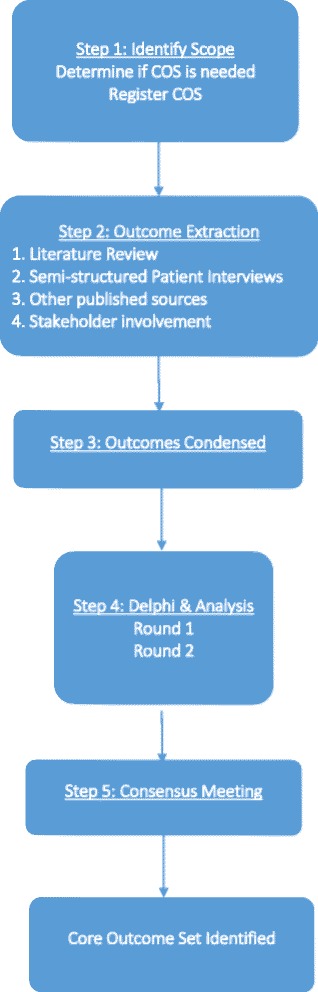
Flowchart of the study design

The development of this COS adheres to the recommendations provided by the COMET and CSG-COUSIN initiatives [14, 21]. Reporting conforms to the SPIRIT (Standard Protocol Items: Recommendations for Interventional Trials) checklist (see Additional file 1). Figure 1 provides a brief overview of our study design.

### Scope of this COS

This COS is intended as the global/international standard for clinical trials examining the efficacy of various treatments for rosacea as evaluated by physicians, patients, and others invested in its results. The COS to be developed will not be population-specific and may be applied to individuals of all ages, genders, skin types, races, and ethnicities. Similarly, all rosacea-specific interventions, including topical, systemic, and laser therapy, may be evaluated using the outcomes generated from this study.

### Identification of outcomes

The list of outcomes currently reported will be generated over four phases.

- First, a systematic literature review will be performed to extract outcomes assessed in published randomized controlled trials. Cochrane reviews will also be examined for outcome inclusion
- Second, patient interviews will be conducted to determine patient-centered outcomes which should be assessed
- Third, other sources, such as clinical trials registries and rosacea educational and treatment brochures, will be reviewed to ensure all outcomes have been documented
- Prior to the development of a final list of outcomes, stakeholders will be invited to provide insight into further outcomes that they would like included

### Literature review

A systematic literature review using PubMed, Embase, CENTRAL, and CINAHL will be conducted using search terms related to rosacea. Included studies will be randomized controlled trials of rosacea, regardless of study quality or bias. Duplicate studies which cross over from different databases will be removed and included only once. Each article will then be extracted. Authors, year of publication, source of funding, and treatment vehicle will be documented among other study characteristics. In terms of study design, items such as length of follow-up, treatment duration, results, outcomes, and outcome measures will be noted. The long list of outcomes extracted from published studies will then be placed into appropriate domains by two investigators.

Domains will be determined based on the outcomes extracted and will encompass treatment efficacy, adverse events, and quality of life. Outcomes that are very similar or synonymous in nature will be collapsed and listed only once. If two outcomes are the same, the shorter version of the name will be applied. Subcategories will be listed appropriately to further stratify a domain. If several similar entries are combined, the constituents will be listed in brackets after the new category. Combining and collapsing of outcomes, while necessary, will be performed in moderation to preserve the content and significance of distinct outcomes.

### Patient-centered outcomes

In order to encompass patient-centered outcomes, a semistructured interview will be conducted to explore the domains identified in the literature review as well as other potential patient identified outcomes. Approximately 10-15 patients with rosacea will be interviewed. A global context will be provided by including participants both in the United States and internationally. Informed consent will be obtained from each participant. Open-ended questions will allow for patient expression of relevant outcomes, which may provide identify outcomes not noted in established quality of life questionnaires or prior studies. Interviews will be audio-recorded, transcribed, and coded to allow accurate and complete capturing of outcomes mentioned. Through the addition of outcomes ascertained during the interviews, we hope to provide a more complete account of issues important to patients.

### Additional sources

Examination of other published sources, including clinical trials registries and Cochrane reviews, will be conducted to gather outcomes related to rosacea. Pamphlets and brochures describing treatments and reported outcomes will be extracted, and outcomes detected will be included in the final list of outcomes.

### Stakeholder involvement

Stakeholders, or those invested in the development of a COS in rosacea, will also be included in the decision process (Table 1). Dermatologists, drug and device safety regulators (e.g., FDA, EMA), patient support groups (e.g., National Rosacea Society), pharmacologists, pharmacists, and industry scientists associated with drugs and devices for treatments of rosacea are potential members who can provide input regarding what outcomes that they feel should be represented. Nurses, physician assistants, and other health care practitioners may also be included to enhance further discussion.

**Table 1:** Summary of stakeholder involvement

Key stakeholders	
Physicians (including dermatologists, international providers, physicians of other health care fields)	
Patients	
Drug and device safety regulators (e.g., FDA, EMA)	
National Rosacea Society/support groups	
Pharmacologists/pharmacists	
Industry scientists	
Nurses, physician assistants, or other health care providers	

*EMA* European Medicines Agency, *FDA* US Food and Drug Administration

### Potential outcomes

The long list of outcomes obtained from the steps described above will then be examined by the Steering Committee, composed of four dermatologists: Drs. Murad Alam (Northwestern University), Ian A. Maher (Saint Louis University), Joseph F. Sobanko (University of Pennsylvania), and Todd V. Cartee (Pennsylvania State University). These members may add or remove outcomes as they deem suitable to be included in the list prior to the Delphi process. The Steering Committee members will not join in the Delphi process, but members will be invited to participate in the final consensus meeting.

### Delphi overview

Delphi surveys have been used in prior COS research [22]. Surveys can be conducted online through the use of specialized software. The Delphi process involves a series of rounds of data collection and analysis to condense the opinions of individuals into a group consensus. Responses to each round are collected, analyzed, and then redistributed to participants in successive rounds. We plan to conduct two Delphi rounds prior to the consensus meeting.

### Participants

Two homogenous groups composed of patients or physicians, respectively, will participate in the Delphi exercises. Groups will consist of approximately 30 individuals to provide a greater diversity of input and account for potential dropouts. A global context will be provided by including patients from both the United States and internationally; physicians from across the country and internationally will be included in the other group. Prior to the exercise, details of the COS will be summarized in plain language. Demographic and occupational information, including years of experience, field of interest, and position, will be obtained. Consent will be assumed if participants complete the questionnaire. Participants will have 3 weeks to complete the online survey with email reminders at the 1- and 2-week marks. For each round, the number of participants invited and those who completed the surveys will be documented and attrition assessed.

### Delphi rounds

In the first Delphi, the complete list of outcomes gathered from the literature review, patient interviews, stakeholder interaction, and other sources will be presented for rating. Outcomes will be listed randomly after each round to avoid any influence the display order may have when scoring. Using a scale devised by the Grading of Recommendations Assessment, Development and Evaluation (GRADE) working group, participants will score each outcome on a scale from 1 to 9 with “9” being critically important and “1” being not very important [23]. For the first round, the additional option of “10” will be available if participants are unsure of its need for inclusion.. Participants will be asked to focus on ranking the most valued outcomes high and excluding outcomes of lesser importance. They will also have the option to add outcomes to the list that they feel should be included. All outcomes will be carried to the subsequent round.

Descriptive statistics will be used to analyze the data from the two groups. Responses from both stakeholder groups will be summarized and fed back to the participants after the first round, allowing participants to change their score in light of others’ insights. At the same time, participants will be asked to identify new outcomes and determine if outcomes should be combined. New outcomes will be added to the list for the next round if two or more participants suggest its inclusion. Any uncertainties will be directed to the Steering Committee for adjudication.

In round 2 of the Delphi exercise, participants will again score the outcomes on a scale from 1 to 9, following the same format as the previous Delphi exercise. The end result of the Delphi should consist of a more simplified set of outcomes that will be further examined at the consensus meeting.

### Consensus meeting

Prior to solidifying a core set of outcomes, a face-to-face consensus meeting will be held to discuss the results of the Delphi rounds. An independent facilitator will chair the meeting to finalize the outcomes. Physicians, patients, and other stakeholders will be invited to the meeting to provide insight on the process. Results from each round of the Delphi survey will be presented. In terms of consensus, if 70 % of participants rank the outcome 7, 8, or 9 with less than 15 % scoring it 1–3, the outcome will be *retained* in the consensus pool [24]. Outcomes will be *removed* from the consensus list if 70 % or more of the participants rank the outcome 1–3 and less than 15 % rank the outcome 7, 8, or 9.

Feedback regarding the consensus-derived set of outcomes will then be elicited with the assistance of a trained moderator. Using live polling software, items will anonymously be voted “Yes” or “No” for inclusion into the final core set of outcomes. By the end of the meeting, the goal is to create a core set of outcomes which can be agreed upon by all stakeholders, patients, and physicians.

### Core outcome measures

Once a COS has been developed, the Harmonizing Outcome Measures for Eczema (HOME) roadmap will be utilized for developing or selecting a core set of measures to track the outcomes selected [21]. Initial steps include identifying outcome domains and current instruments used to measure those specific domains. Special focus will be directed at systematically identifying relevant outcome measurements through a systematic review covering multiple databases. Quality of the studies will be assessed by rating their validity, reliability, responsiveness to change, and interpretability. The COnsensus-based Standards for the selection of health Measurement INstruments (COSMIN) framework will be utilized for guidance.

In order to determine which measurements are suitable per outcome domain, a consensus meeting with key stakeholders, patients, and clinicians will be held [21]. Results from the systematic review will be provided to guide discussion. Attendees will then judge the measures based on how valid, reliable, and feasible they may be for assessing each core outcome domain. If evidence for a particular measurement instrument is inadequate, it may be necessary to develop a new instrument. At the end of the consensus meeting, relevant stakeholders will vote to determine which measures should be included. An outcome measurement instrument should be recommended for each core outcome domain.

## Discussion

There is currently no COS relevant to clinical trials of rosacea. With a lack of standardization in outcomes assessed, the potential for reporting bias exists. Further, selection of outcomes is crucial for properly comparing and evaluating the effectiveness of different treatment interventions.

The proposed COS for rosacea aims to reduce the inconsistency of outcomes and outcome measurements across relevant trials. Through the use of COSs, we hope to hasten refinement and adoption of therapies for rosacea that address factors important to key stakeholders, particularly patients and physicians.

## Trial status

The development of the COS is active and ongoing in its initial phase of outcome extraction.

## Additional file

Additional file 1: SPIRIT checklist. Completed checklist of the study protocol for the development of a core outcome set. (DOCX 47 kb)

## Abbreviations

COMET: Core Outcome Measures in Effectiveness Trials
COS: core outcome set
COSMIN: COnsensus-based Standards for the selection of health Measurement INstruments
GRADE: Grading of Recommendations Assessment, Development and Evaluation
CSG-COUSIN: Cochrane Skin Group - Core Outcome Set Initiative
EMA: European Medicines Agency
FDA: US Food and Drug Administration
HOME: Harmonizing Outcome Measures for Eczema
